# Assessing the Influence of Cardinality Constraints on the Simultaneous Optimization of Truss Sizing, Shape, and Topology

**DOI:** 10.3390/ma18071457

**Published:** 2025-03-25

**Authors:** Nenad Petrović, Nenad Kostić, Nenad Marjanović, Ružica R. Nikolić, Robert Ulewicz

**Affiliations:** 1 Faculty of Engineering, University of Kragujevac, 34000 Kragujevac, Serbia; npetrovic@kg.ac.rs (N.P.); nkostic@kg.ac.rs (N.K.); nesam@kg.ac.rs (N.M.); 2 Research Centre, University of Zilina, 01026 Zilina, Slovakia; 3 Department of Production Engineering and Safety, Czestochowa University of Technology, 42-201 Czestochowa, Poland

**Keywords:** truss optimization, cardinality, Euler buckling, optimization constraints, sizing, shape, topology optimization

## Abstract

The influence of cardinality constraints on the simultaneous optimization of truss sizing, shape, and topology, when used for weight minimization, is explored in this study. By integrating precise cardinality constraints, the implications for achieving global optimal solutions for different numbers of different cross-sections were investigated. This research underscores the significance of these constraints in enhancing the practical applicability of optimization outcomes, particularly in complex structural configurations where traditional approaches may lead to excessive requirements of numerous different cross-sections. Comprehensive experimentation and comparative analysis, across various standard and practical truss examples, demonstrate the effectiveness of cardinality constraints in guiding optimal design configurations. Notably, the presented findings reveal a trend in weight savings, depending on the number of different cross-sections used relative to global optima, displaying the utility of this constraint in achieving practical and efficient designs. Case studies on a produced roof truss underscore the applicability of this approach in practical engineering scenarios. They offer insights into the optimal design configurations for problems that do not allow for drastic changes due to their restrictive design mandate. This research is part of continued advancements in truss optimization methodologies, with implications for promoting sustainability and cost-effectiveness in structural engineering practice. By elucidating the role of cardinality constraints in shaping the optimal design solutions, this study should contribute to the broader discourse on efficient structural design strategies.

## 1. Introduction

In the field of truss structural design, optimization has the greatest potential to contribute to the efficiency and sustainability of the final product. To this end, numerous advancements have been made over the years, since the optimization started being researched for these purposes. The design can be optimized for sizing, shape, or topology, as well as a sequential or simultaneous combination of any of the three types. The use of discrete variable sets has allowed for optimized structures to use commercially available cross-sections of bars, though there are still publications using continuous sizing variables [1]. Basic static constraints, such as tension and compression loading limits and maximal nodal displacement resulting from loading, have been used practically since the beginning.

More recently, the dynamic constraints, which vary with each iteration, have become prevalent as computational capabilities and heuristic optimization methods allowed for faster operation and easier navigation of the search space. The inclusion of buckling constraints, as one of these dynamic constraints, has proven in [2,3,4,5,6] to create truss structures which are practically applicable or at least closer to application.

The majority of solutions presented in the aforementioned papers, though of minimal weight, present impractical solutions in terms of the large number of various cross-sections used. For real-world applications, trusses, depending on complexity, are designed using a few different cross-sections. Using the conventional design methods might not always yield the lowest possible weight of a structure as they are labor and time-intensive and rely on experience, but the added simplicity of using only a few different profiles eases the calculation, sourcing, and assembly, decreases wasted stock, and has many other secondary benefits.

An approach that can help in practical applications was used by the authors in [7]. Therein, the design of a structure was optimized to use the stock of reclaimed elements. This iterative process first assigns elements and conducts topology optimization, and then geometry optimization follows to best fit the system geometry to the length of the assigned stock elements.

Another approach is through the limiting of the number of different cross-sections used in the optimization of trusses, which is performed using cardinality constraints. Researchers in [8,9] used the genetic algorithm (GA) to optimize truss structures for sizing and simultaneous sizing and shape. They used an encoding method for automatic variable linking, which does not allow for solutions that do not meet the cardinality constraint, eliminating the need for a penalty function for this constraint. The same approach was used in [10] for simultaneous sizing and layout optimization and in [11] for sizing optimization. The authors of [12] analyzed the impact of minimizing the number of different cross-sections as a multi-objective optimization goal. They used 16 evolutionary algorithms to achieve these conflicting goals and presented their findings for 10-, 200-, 582-, and 942-bar trusses and a proposed 336-bar truss example. An ant colony approach to multi-objective structural optimization was used in [13] where the cardinality constraint was built into the algorithm. The authors minimized the weight and nodal displacement using a two-step construction process for assigning design variables according to defined cardinality. In [14,15], the authors also used the GA to optimize the same aspects of planar and spatial trusses, using a different approach to constraining cardinality, which allows for solutions to be penalized for not meeting cardinality criteria. That approach for constraining cardinality was used in this research as well to show its merits in the simultaneous optimization of sizing, shape, and topology. The addition of topology optimization further complicates the problem compared to previous applications, as each iteration has the possibility of having a different number of bars used in the configuration. In the process of optimization, this issue has been handled by the newly adapted solution in the original software in Rhinoceros 6’s Grasshopper and Karamba3D 2.2.0 which maintains initial annotations from the example’s numbering scheme to allow the algorithm to use generational data in subsequent iterations.

The main issue, which was overcome in this research, is the transfer of previous generational data to subsequent iterations, which is necessary for implementing a combination of sizing and topology optimization. This has been overcome in the approach by referencing each bar according to its initial layout nomenclature. The initial idea for this was included in these authors’ previous paper [15]; however, this system was not implemented there, since there was no change in topology. In that phase of research, the system was still being developed and recognized as a necessity for including topological optimization. This paper presents this change, as well as the expected benefits of implementing cardinality constraints on both test problems and a real-world example.

This research also expands on the previous research showing how this approach can be used on a practical roof truss with a very limited optimization space.

## 2. Simultaneous Truss Sizing, Shape, and Topology Optimization

In the field of truss structural optimization, sizing optimization considers cross-sections as variables. This research looks at cross-section variables as a discrete set of values to achieve applicable results. Shape optimization considers the location of nodes as variables. For the purposes of varying the location of nodes, planar or special coordinates are continually variable with a granularity of 1 mm tolerance. Topology optimization considers the inclusion of bars in the structure as variables. In this paper, each structural example undergoes scrutiny to determine which bar elements could be potentially removed and which are indispensable in any configuration. Specifically, bars are considered non-variable if their absence would lead to an absence of stable loading or support locations in the required node. Each bar from the initial layout maintains its initial annotation in the optimization in order to ensure subsequent generations can use the previous generation’s data to further the search for a global optimum. This is performed through annotation assignment to bars separate from the iteration set, which matches the initial layout numbering scheme to each new generation. Without this step, each generation would assign cross-sections chronologically instead of matching it to the node pair between which the bar is located, making each generation, basically, a random instance.

The objective function of most research in the field [2,11,12,13,14,15], including this one, is to find the combination bars connected in specific locations with assigned cross-sections, which give a minimal weight, while subjected to adequate constraints. For the purposes of this study, the material of the beams was not considered as a variable, though this is also a possibility. The objective function is given as follows:

(1) $$\min W=\overset{n}{\underset{i=1}{\sum }}\;\;\rho_{i}\;A_{i}l_{i}\;$$

where *W* is the weight of the truss (not including connections and supports), and *n* is the number of used truss bar elements. Since the topological optimization is considered in this study as a part of the structural optimization process, *n* is also a variable. The area of the *i*-th element cross-section is *A_i_*, and *l_i_* is the length of the *i*-th element.

### 2.1. Stress and Displacement Constraints

Optimization constraints ensure structural functionality under the predefined loading conditions while maintaining the structural integrity within the elastic zone. The stress constraint for bar elements is as follows:

(2) $$\frac{\left|\sigma_{i}\right|}{\sigma_{max}}-1\leq 0\;\mathrm{for}\;i=1,2,\ldots ,n$$

where *σ_i_* is the stress of the *i*-th element, and *σ_max_* is the maximum allowable stress. The displacement constraint ensures that the displacement of nodes under load does not exceed the predefined values as follows:

(3) $$\frac{\left|u_{j}\right|}{u_{max}}-1\leq 0\;\mathrm{for}\;j=1,2,\ldots ,k$$

where *u_j_* is the displacement of the *j*-th node, *u_max_* is the maximum allowable displacement in any direction, and *k* is the number of nodes.

### 2.2. Euler Buckling Constraints

Compressed elements must also be tested for the buckling stress (4). Euler buckling constraints also consider the minimum area moment of inertia, which is why the results of these examples are only useful for the specific profile shape with which they are optimized.

The Euler buckling is added to obtain results that can be applied in practice. Due to the change in cross-sections, the iterative change in the moment of inertia also changes the Euler critical buckling constraint in each iteration (5). This constraint is, therefore, considered to be a dynamic constraint. Its addition, it significantly increases the complexity of the optimization problem, as follows:

(4) $$\frac{\left|\sigma_{Ai}^{\;comp}\right|}{\sigma_{crit.\;\;i}}-1\leq 0\;\;\mathrm{for}\;\;i=1,2,\ldots ,n\;\;\;\mathrm{where}\;\;\;\sigma_{Ai}^{comp}=\frac{F_{Ai}^{comp}}{A_{i}}\;\;\;\mathrm{and}\;\;\;\sigma_{crit.\;\;i}=\frac{F_{crit.\;\;i}}{A_{i}}$$

where *σ_Ai_* is the axial compression stress of the *i*-th bar element and *σ_crit. i_* is the critical buckling stress of the *i*-th bar element. Euler critical load is used in this research since the stress comparison uses the same area to determine any given element’s compression and critical stress, as follows:

(5) $$\frac{\left|F_{Ai}^{\;comp}\right|}{F_{crit.i}}-1\leq 0\;\;\mathrm{for}\;\;i=1,2,\ldots ,n\;\;\;\;\mathrm{where}\;\;\;F_{crit.\;\;i}=\frac{\pi^{2}\;\cdot E_{i}\cdot \;I_{i}}{l_{i}^{2}}$$

where *F_crit.i_* is the Euler’s critical load of the *i*-th element, *E_i_* is the *i*-th element’s modulus of elasticity, *I_i_* is the minimum moment of inertia of the *i*-th element’s cross-section, *l_i_* is the length of the *i*-th element, and $F_{Ai}^{\;comp}$ is the axial compression force.

### 2.3. Minimal Element Length Constraint

In this study, aside from the continuous variable set limits for node coordinates, an additional shape optimization constraint on the minimum element length is incorporated to prevent the occurrence of overly short elements in solutions, which could pose impractical challenges during implementation. It is not uncommon to find optimal solutions with zero, or near zero element lengths, which satisfy all other constraints, but in practice would be impossible or tedious to make, thereby negating the effects of the savings achieved through the weight optimization. Determining the constraint value for each example is guided by established design standards, literature references, or empirical experience. The formulation of this constraint is delineated as follows:

(6) $$\frac{l_{i}}{l_{\min}}-1\leq \;0=\;\;\;\;\;\mathrm{for}\;i\;=1,2,\;\;\ldots ,\;\;n\;\;\;\mathrm{where}\;\;\;l_{i}=\sqrt{{\left(x_{bi}-x_{ai}\right)}^{2}+{\left(y_{bi}-y_{ai}\right)}^{2}}$$

where element length *l_i_* is from the set of used elements in any iteration, 1 to *n*. Each bar element has nodes *a_i_* and *b_i_* at each end, which are defined as ($x_{ai},y_{ai}$) and ($x_{bi},y_{bi}$). If a maximum element length constraint was required, the same approach could be employed. Nevertheless, the predefined limits of the node coordinate variable set inherently define a maximum length.

In this study, a uniform penalty function is applied to all the constraints. This function involves multiplying any non-compliant results by a substantial factor to penalize instances where one or more constraints are not satisfied.

### 2.4. Cardinality Constraint

**Figure 1:**
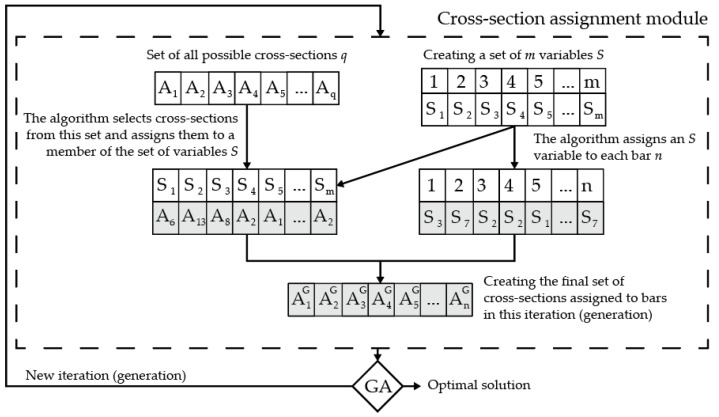
A schematic overview of how cross-section assignment works with the cardinality constraint.

This research is an expansion of the works [8,9,14,15], which implemented cardinality constraints to further increase the applicability of optimization results in the field of trusses. To implement this constraint, the entire process of cross-section selection and assignment has been changed from the approaches that do not use this constraint. The mathematical expression for constraining the number of different cross-sections, used in a solution, is given as

(7) $$\frac{m}{m_{\max}}-1\leq 0\;\;\;\mathrm{where}\;\;m=\;|\{A_{1}^{G},A_{2}^{G},A_{3}^{G},\ldots ,A_{n}^{G}\}|\;\;\;\mathrm{and}\;\;\;m\leq m_{\max}\leq n$$

where *m* is the cardinal of the set of used cross-sections for the proposed solution, *m_max_* is the maximal allowed number of different cross-sections in any given solution, and $A_{n}^{G}$ is the cross-section assigned to the *n*-th element. This constraint is applicable for all types of cross-sections, as the set of used cross-sections needs to include or be linked to all the necessary geometrical information about the used cross-section to allow for all of the necessary calculations. Using a two-step approach with variables being reassigned to a newly selected set, the authors of this study have developed an original solution in Rhinoceros 6’s Grasshopper and Karamba3D 2.2.0. The process of implementing the constraint from expression (4) is created within a module in the optimization process, as shown in Figure 1. The chosen optimization algorithm is a genetic algorithm (GA) due to its availability in the software, its possibility to handle this type of problem, and its comparability with relevant research. The algorithm has been around for a very long time, but it is still relevant and used in research to date [14,15,16].

The starting discrete set of all the possible cross-sections that could be used for the given example contains all the necessary geometrical information about cross-sections from 1 to *q*. At the same time, a set of *m* variables is created according to the set value *m_max_*. The algorithm then assigns the set *S* of m values to a cross-section from the set of all the possible cross-sections, while the same set *S* is also assigned to a bar element. The resulting set has *m* different cross-sections assigned to *n* elements. The variables in the set *S* is always referencing the same bar with the same number from the initial layout throughout the optimization process to match sizing optimization genetic variations to the corresponding bar from the previous iteration.

For this research, to determine the influence of each set value of *m_max_*, even beyond the global optimal number, the inequality in expression (4) is set to *m* = *m_max_* ≤ *n*. In regular applications, as long as the *m_max_* value is set to less than the global optimum number of cross-sections the optimization, the process will provide a solution with *m_max_* different cross-sections. The addition of optimizing topology simultaneously with other aspects of the structure also influences the results as the number of used cross-sections is limited on the maximum end by the number of used bars in a particular solution.

## 3. Test Examples

Some of the most frequently used test examples found in the literature use the full cross-sections. This is a remnant from the time when buckling constraints were not considered, so the cross-section’s shape was unimportant and just the areas were important as they could be used to size an equivalent area cross-section of any shape. This is also why, in this research, the results for optimal cross-sections are given as areas instead of diameters. In reality, buckling is an important sizing factor for compressed elements. To present the influence of using cardinality constraints, the use of the constraints is shown on standard test examples of 10-bar, 17-bar, and 25-bar truss examples commonly found in the literature, which use full circular cross-sections to present results comparable to those in the literature. Additionally, a practical example of a roof truss that uses hollow square profiles is presented using the same constraint types.

All the examples incorporate dynamic Euler buckling constraints to address compressed elements, ensuring that the optimal truss configurations remain within the elastic range. Each example introduces a new cardinality constraint, specifying the exact number of allowable cross-sections for optimization runs. The use of precise numbers is aimed at identifying the optimal values for varying cardinalities and discerning trends in these changes. This constraint is proposed to establish a maximum rather than a precise count of cross-sections, as demonstrated here. Optimization was conducted for each cardinality level, both below and above the optimal number of cross-sections. Instances exceeding the optimal count were included solely for the trend observation and are not practically beneficial.

### 3.1. Planar 10-Bar Truss Problem

**Figure 2:**
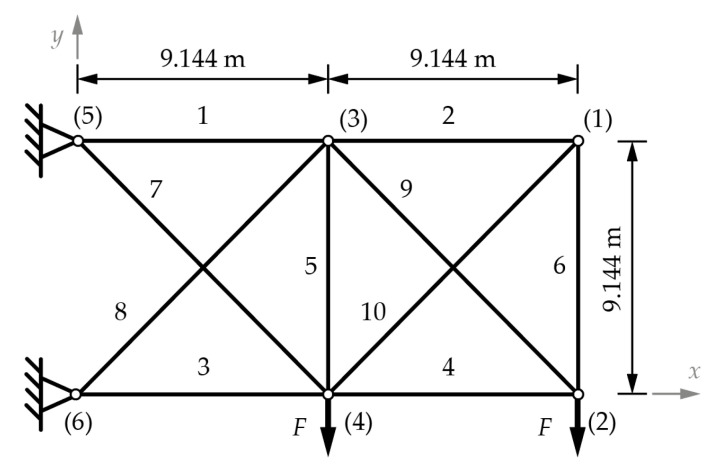
The 10-bar truss with dimensions and labeled nodes ((1) to (6)) and labeled bars (1–10) [14].

The planar 10-bar truss is the most commonly used example for testing the new truss optimization methods and approaches. The layout of the initial structure is shown in Figure 2 using aluminum 6063-T5 bars, with a Young modulus of 0.7·10^5^ MPa and a density of 2700 kg/m^3^. The application of a point load with a magnitude of *F* = 444.82 kN in the negative y-direction is imposed upon nodes (2) and (4).

Constraints include a maximal displacement under load tolerance of ±0.0508 m for all the nodes in every direction, maximal axial stress within the range of ±172.37 MPa for all the bars, and adherence to Euler buckling criteria for all compressed bars. The chosen set of cross-sections comprises 50 distinct diameters, ranging from 3 mm to 125 mm, as follows: 3, 4, 6, 6.5, 7, 7.5, 8, 8.5, 9, 10, 11, 12, 12.5, 14, 15, 16, 17.5, 18, 19, 20, 22.5, 25, 27.5, 28, 30, 31.5, 32.5, 35, 37.5, 40, 42.5, 45, 47.5, 50, 52.5, 55, 57.5, 60, 62.5, 65, 70, 75, 80, 85, 90, 95, 100, 110, and 125 mm. The topology is limited to maintaining at least two bars in nodes (3) and (4) to ensure the possibility of loading, and any optimization iteration can only remove one of the bars from the set {1, 3, 7, and 8}. The positional coordinates of nodes (3) and (4) are treated as variables within the confines of the initial geometry. Specifically, the *x* and *y* directions allow for variation within the specified bounds: ±9.144 m in the *x* direction for nodes (3) and (4) and −9.144 m in the *y* direction for both nodes.

### 3.2. Planar 17-Bar Truss Problem

**Figure 3:**
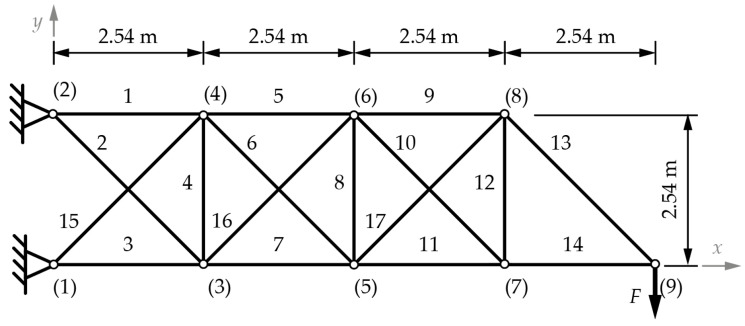
The 17-bar truss with dimensions and labeled nodes ((1) to (9) and bars (1–17)) [14].

This example uses steel with a Young modulus of 2.1 ∙ 10^5^ MPa and 7400 kg/m^3^ density for all 17 bars. The load applied to node (9) is 444.82 kN in the −*y* direction. The reason for selecting this example is that it does not include a stress constraint other than for the Euler buckling of only the compressed bars. Displacement under load is, however, constrained to ±0.0508 m in both *x* and *y* directions for nodes (3) to (9). The initial layout of this problem is shown in Figure 3.

The discrete set of variables for cross-sections and their geometry are the same as in the previous example, despite the change in material. Coordinates for nodes (3) to (8) can all vary from 0 to 10.16 m in the *x* direction and from −2.54 to 5.08 m in the *y* direction with respect to the initial configuration. Only the *y* component of the coordinates for node (9) can vary from 0 to 2.54 m from the initial configuration. Topology optimization is limited not to allow the exclusion of bars 13 and 14. Any optimization iteration can only remove one of the bars from the set {1, 2, 3, and 15}. These limitations for the bar removal reduce the search space since it is obvious that without this limit, there will be iterations, which are mechanisms.

### 3.3. Spatial 25-Bar Truss Problem

**Figure 4:**
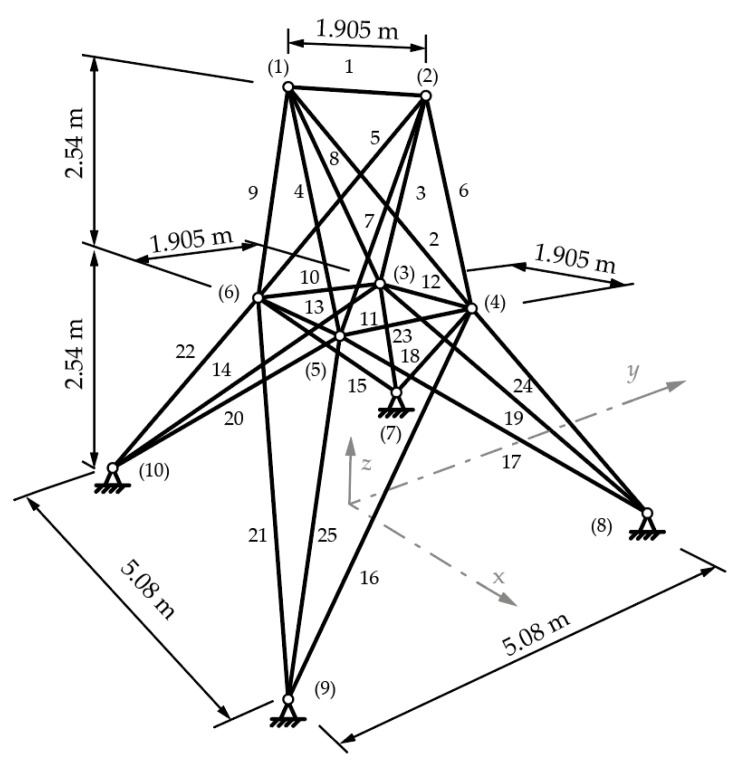
The 25-bar truss with dimensions and labeled nodes ((1) to (10)) and bars (1–25) [14].

The 25-bar truss is a spatial problem that uses the same cross-section variables and material as the planar 10-bar truss. This example has bars grouped into sets to ensure that each bar group has the same cross-sections assigned to all the bars in that group. These eight sets consist of bars, which can be seen in Figure 4 and are grouped as follows: 1 {1}, 2 {2–5}, 3 {6–9}, 4 {10–11}, 5 {12–13}, 6 {14–17}, 7 {18–21}, and 8 {22–25} [14]. Forces applied to this example are given by (*x*, *y*, *z*) components for nodes (1) (4.448, −44.48, −44.48) kN, (2) (0, −44.48, −44.48) kN, (3) (2.224, 0, 0) kN, and (6) (2.669, 0, 0) kN.

The structure is constrained to a stress limit of 40 kN in all the bars, and Euler buckling limits are used for bar groups where at least one element is compressed. The displacement of nodes under load is constrained to ±0.009 m in all directions for all nodes. Shape variables are created in such a way as to maintain the loading directions but to allow for variation in the geometry. The shape variables (node coordinates) are continuous with increments of 1 mm. The limits for node variables according to node are as follows: 0.508 m ≤ *x*_4_, *x*_5_, −*x*_3_, −*x*_6_ ≤ 1.524 m; 1.016 m ≤ *y*_3_, *y*_4_, −*y*_5_, −*y*_6_ ≤ 2.032 m; 2.286 m ≤ *z*_3_, *z*_4_, *z*_5_, *z*_6_ ≤ 3.302 m; 1.016 m ≤ *x*_8_, *x*_9_, −*x*_7_, −*x*_10_ ≤ 2.032 m; and 2.540 m ≤ *y*_7_, *y*_8_, −*y*_9_, −*y*_10_ ≤ 3.556 m.

### 3.4. Roof Truss Problem

**Figure 5:**
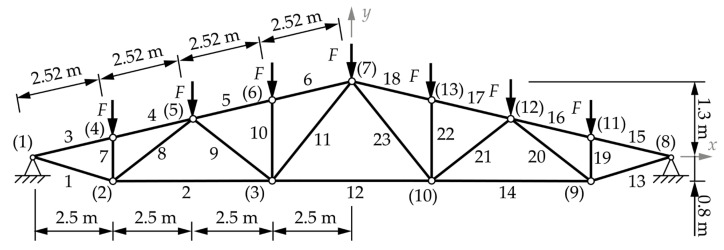
Roof truss initial topology layout with dimensions and labeled nodes ((1) to (16)) and bars (1–23) [17].

**Figure 6:**
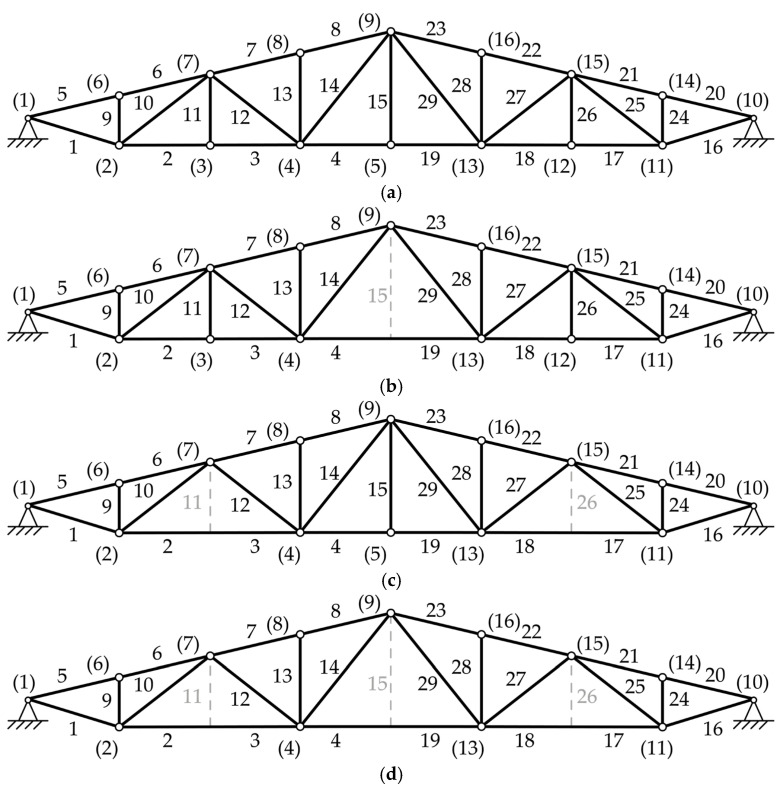
Roof truss topology configurations. (**a**) Layout 1 as seen in Figure 5, (**b**) Layout 2 without bar 15 with rigid connection between bars 4 and 19, (**c**) Layout 3 without bars 11 and 26 with rigid connection between bars 2 and 3 and bars 17 and 18, and (**d**) Layout 4 without bars 11, 15, and 26 with rigid connection between bars 4 and 19, bars 2 and 3, and bars 17 and 18.

The practical roof truss is a symmetrical structure about the *y-axis*. This example is not suitable for topological optimization in the conventional sense. This is due to the fact that removing any bar from the initial configuration, as seen in Figure 5, would result in an unstable structure if nodes are considered as joints in the calculation process. For this reason, this example considers four layouts of topology, as shown in Figure 6, to show all the possible stable topologies without removing loaded or support nodes [17]. This means that all the layouts were optimized only for sizing and shape simultaneously. All the profiles are S235JRG2 steel hollow square sections (HSS), with a Young modulus of 2.1 ∙ 10^5^ MPa and a density of 7850 kg/m^3^. The profiles used, along with their moments of inertia, are given in Table 1.

The structure is divided into nine different groups of bars, which are symmetrically arranged. These groups are used to assign the same cross-section to a whole group of bars. Grouping is conducted so that there are no changes in cross-section along a straight line of elements. The bars are grouped as follows: bars 1 to 4 with 16 to 19, bars 5 to 8 with 20 to 23, bar 9 with 24, bar 10 with 25, bar 11 with 26, bar 12 with 27, bar 13 with 28, bar 14 with 29, and bar 15.

Compression and tension stress limits are set to 180 MPa for all bars, along with Euler buckling constraints for compressed elements, as well as a maximum allowed displacement under load of ±0.036 m for all nodes in the *x* and *y* directions. Shape constraints are grouped to ensure that the symmetry is maintained for each iteration. The coordinates are constrained to 5.5 ≤ −*x*_2_ = *x*_11_ ≤ 8.5, 3 ≤ −*x*_3_ = *x*_12_ ≤ 7, 0.5 ≤ −*x*_4_ = *x*_13_ ≤ 4.5, and 0 ≤ *y*_2–5_ = *y*_11–13_ ≤ −0.8 (given in meters).

## 4. Results

The presented optimization results for all examples are the best of 10 repeated processes, always starting from the analytical solution of the initial example configuration. In the case of the 10-bar, 17-bar, and 25-bar trusses, the analytical solution was dimensioned according to the most loaded compressed bar of the initial shape and topological layout, and the same profile was used for all bars of that example. In the case of the roof truss, the production dimensions were used. All examples are optimized with a forced constraint of cardinality greater than their respective global optima to show a trend and prove that the global optima does not use more different cross-sections. For example, with 10 and 25 bars, the maximum number is set to one less than the initial configuration number of bars (bar groups) to ensure that topology optimization is employed as well. For the 17-bar and roof examples, the smaller numbers were chosen as their global optima, which used a smaller number of different cross-sections than the maximum number of possible elements.

The parameter for termination of the genetic algorithm was set to a maximal stagnant population of 50. Each generation was set to a population size of 50, maintaining the top 5% onto the next generation and inbreeding of 75%. The same optimization parameters were used for all the examples. It should be noted that the results presented here can be obtained using any other heuristic method of optimization. The main reason for using the genetic algorithm (GA) was for its availability, widespread use, and reliability. Any other heuristic algorithm can be implemented. This research does not focus on algorithm speed and performance but rather on the possibilities of bringing optimization closer to application in the design process.

Table 2 shows the optimum cross-sections and weights for the 10-bar truss sizing, shape, and topology optimization problem for cardinality constraints from 1 to 9. In this example, the optimal solution uses five different cross-sections. The coordinates of points (1) and (3) are given in Table 3 for each optimal solution according to the solution. Solutions with cardinality constraints ranging from 1 to 4 do not include node (1) in their optimal configurations, as evident from Table 2, since these solutions do not utilize bars 2, 6, and 10.

**Figure 7:**
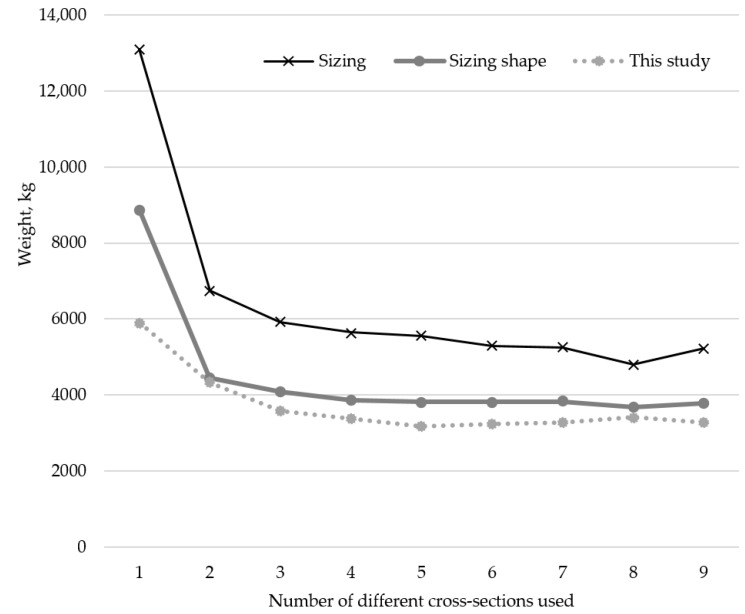
Comparison of results for sizing optimization [14] and sizing shape optimization [15] to results from this research for different numbers of cross-sections of the 10-bar truss problem.

A comparison of the trend of optimal weight results, depending on the number of different cross-sections used for the 10-bar truss example, depending on what aspects of the truss are optimized, is shown in Figure 7. The results from [14] are shown as sizing results and simultaneous sizing and shape optimization results are shown from [15].

Table 4 shows the optimal cross-sections and weights for the 17-bar truss sizing, shape, and topology optimization problem, for cardinality constraints from 1 to 10. In this example, the optimal solution uses six different cross-sections. The coordinates of points (3) to (9) are given in Table 5 for each optimal solution, according to the solution.

**Figure 8:**
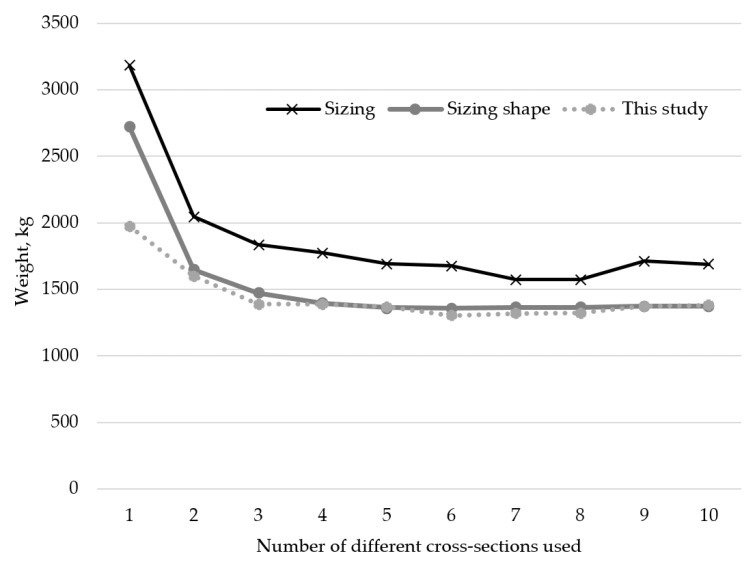
Comparison of results for sizing optimization [14] and sizing shape optimization [15] to results from this research for different numbers of cross-sections of the 17-bar truss problem.

A comparison of the trend of optimal weight results depending on the number of different cross-sections used for the 17-bar truss example depending on what aspects of the truss are optimized is shown in Figure 8. The results from [14] are shown as sizing results, while simultaneous sizing and shape optimization results are shown from [15].

Table 6 shows the optimal cross-sections, according to element groups, and weights for the 25-bar truss sizing, shape, and topology optimization problem, for cardinality constraints from 1 to 7. In this example, the optimal solution uses five different cross-sections in a total of six groups. The coordinates of points (3) to (10) are given in Table 7 for each optimal solution, according to the solution.

**Figure 9:**
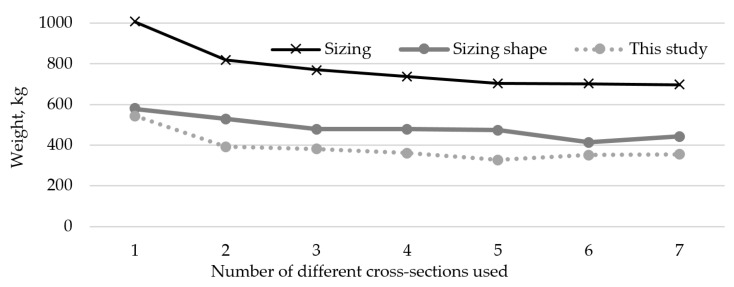
Comparison of results for sizing optimization [14] and sizing shape optimization [15] to results from this research for different numbers of cross-sections of the 25-bar truss problem.

A comparison of the trend of optimal weight results, depending on the number of different cross-sections used for the 25-bar truss example, depending on what aspects of the truss are optimized, is shown in Figure 9. The results from [14] are shown as sizing results, while simultaneous sizing and shape optimization results are shown from [15].

**Figure 10:**
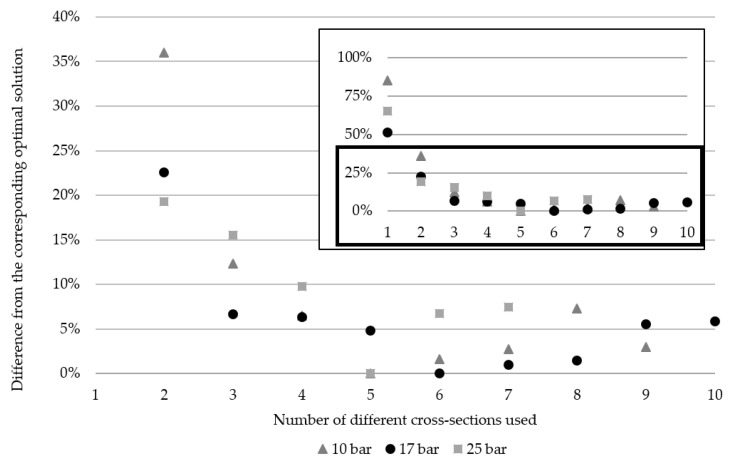
Differences from the corresponding optimal solutions are based on the number of different cross-sections used for 10-bar, 17-bar, and 25-bar examples.

The percentage differences in each cardinality solution, from the respective overall optimal solutions for the 10-bar, 17-bar, and 25-bar truss examples are shown in Figure 10 to illustrate the trend of weight increase as the cardinality constraint moves away from the global optima. The graph displays all the values along with a scaled version of values below 40% to highlight variations more clearly. This adjustment is made to enhance visibility since when the graph spans the 0–100% range, the variations are too close to distinguish the markers.

The analytical solution for the roof truss problem is used for the standing structure from which this example was created. This solution uses three different profiles on the first topology configuration in the following way: 90 × 90 × 6 profile for bars 1–4 and 16–19, 100 × 100 × 4 profile for bars 5–8 and 20–23, and for all other bars, the 50 × 50 × 4 profile. The solution is the work of an experienced structural engineer, which is why the solution is already low in weight. The analytical solution weighs 699.683 kg. To put this into perspective, a solution using the same cross-section for all bars, which is sized according to the most loaded compressed bar, weighs 881.841 kg and uses 140 × 140 × 3 profiles. Furthermore, the specifics of the use case for this problem did not allow for large variations in shape, hindering the possible improvements that a larger variation in the shape could potentially bring. Table 8, Table 9, Table 10 and Table 11 give the optimal solution cross-sections according to the number of different cross-sections used for the four roof topology configurations, along with their respective weights.

Coordinates of nodes (2) to (5) and (11) to (13) are shown in Table 12 for all the topology cases, according to the number of different cross-sections used. The configurations of topology cases 3 and 4 do not include nodes (3) and (12) since these cases have rigid connections between bars 2 and 3 and bars 17 and 18, thereby eliminating the need for nodes in these locations.

**Figure 11:**
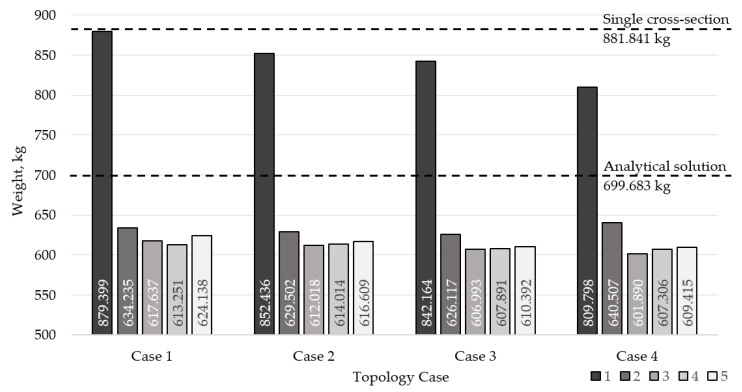
Differences in optimal weight according to topology case and a number of different cross-sections used, with labeled weights of a single cross-section of non-optimized and experience-based analytical solution for topology case 1.

Figure 11 shows the differences in optimal weight according to topology case and the number of different cross-sections used. The weights of single cross-section non-optimized and experience based analytical solutions for topology case 1 are marked, as well.

**Figure 12:**
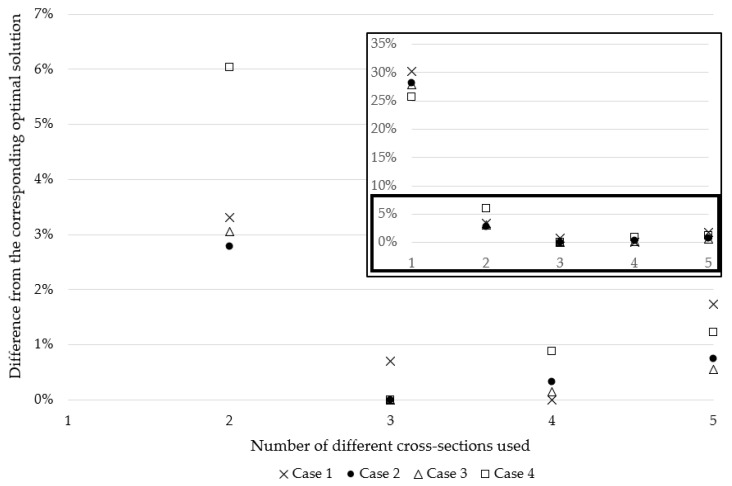
Differences from the optimal solutions are based on the number of different cross-sections used for topology cases 1, 2, 3, and 4.

The percentage differences in each cardinality solution from the respective overall optimal solutions for all roof topology cases are shown in Figure 12 to illustrate the trend of weight increase as the cardinality constraint moves away from the global optima. The graph displays all values along with a scaled version of values below 7% to highlight variations more clearly. This adjustment is made to enhance visibility since when the graph spans the 0–35% range, the variations are too close to distinguish the markers.

## 5. Conclusions

The results presented in this paper are part of extensive research in the field of truss design and optimization. An overall goal in developing new optimization techniques for engineering problems is to achieve applicable results that require zero to very little further designer input. In previous works, sizing and simultaneous sizing and shape optimization were used to test the results of using cardinality constraints to reduce the number of cross-sections an optimal truss solution uses. Here, the complete sizing, shape, and topology optimization were simultaneously implemented to give a perspective on what is possible using this approach and to show how adding complexity improves potential results. To overcome the problem in the topology optimization of carrying over genetic information from iteration to iteration, with the additional complexity of cardinality constraints, this research used a referencing system to maintain the cross-section assignment of sizing optimization relative to the position of the bar that is used (or omitted).

The implementation of cardinality constraints significantly increases the applicability of optimization results in the real world. Experience has shown that global optimal solutions without this constraint use a large number of different cross-sections, especially in more complex structures with wider ranges for sizing and shape variables.

Cardinality constraints for all examples were set to exact values instead of maximal possible values to show the trend after the number of cross-sections used increases past the global optima. This also confirmed that the global optimal solutions were indeed the ones presented here. All the examples were optimized 10 times from the same corresponding initial layouts, and the best solutions are presented here. It should be noted that out of the 10 runs for all examples, most of the results were close to the used results, with only a few runs becoming stuck in local optimal solutions.

To show comparable results, this paper used typical 10-bar, 17-bar, and 25-bar truss examples and compared optimization results to corresponding results from the literature. The main comparison for these examples was between the different numbers of cross-sections used for each example to optimize sizing shape and topology compared to results from the literature of those same examples when only sizing and simultaneous sizing and shape optimization were used. The other comparison is the difference from the global optima for each different number of cross-sections used for each sizing, shape, and topology optimized example of each of the problems.

The difference in using sizing and shape as opposed to sizing shape and topology for the 10-bar example is consistent for 3–7 cross sections used at about 12.5–16.5% and about 38–43% compared to just sizing in the same range, for each corresponding cardinality constrained result. A 2.6% difference between sizing shape and this work is seen when using only two different cross-sections with a total weight still being lower than all sizing optimization results and from the global optima for sizing of using just by less than 10%. A similar trend is observed with the 17-bar truss results when comparing approaches. Namely, sizing and shape compared to this work in the same range of 3–7 differ by approximately 0–5%, while just shape differs by approximately 16–24%. The planar 25-bar truss uses bars grouped by sector, but even here, there is a clear trend of sizing and shape results differing by approximately 20–30% in the range of 2–5 bar groups and sizing results differing by about 50–53% for sizing. These trends indicate that the additional complexity of adding topology optimization does not give drastically different results compared to sizing and shape optimization. In practical terms, however, the decrease in the number of used bars has additional savings in the form of fewer cuts needed to create bars, fewer welds at connections, fewer connections required if topology optimization eliminates the need for a node, etc. An interesting aspect to consider for future work would be how overall surface area is affected depending on weight optimization results to show savings in the amount of needed surface protection, as was performed in [17].

The difference from the global optima for each different number of cross-sections used for each corresponding example of the 10-bar, 17-bar, and 25-bar problems shows a correlation in the weight savings. The observed trend is logarithmic to the point of the global optima, past which the trend is a slight forced increase in weight. In this regard, the results that use only three different cross-sections differ from their respective optima by approximately 6.5–15.5%, while for four cross-sections, this is approximately 6–10%, depending on the example. Three to four different cross-sections are reasonable for this complexity of structures.

Aside from typically used examples from the literature, which use full round cross-sections, a real roof truss with HSS cross-sections was optimized using the same approach. This example differs in the use of topology optimization since it was impossible to remove any bars without creating an unstable truss. This was overcome by optimizing sizing and shape simultaneously for four different topology cases, where the nodes were removed, and bar pairs going through those nodes were replaced by single bars. The results from the roof example are compared case by case, according to the number of different cross-sections used, as well as to a single cross-section solution of the initial layout and the real truss’s dimensions, which was designed using analytical calculation and years of experience of the designer.

When comparing the weight results for each topology case, by the number of different cross-sections used, the distributions are rather similar from case to case, with case 4, which uses the smallest number of nodes, and bars having the lowest optimal weight with three different cross sections. All the cases, regardless of the number of the used cross-sections, have a lower optimal weight than when just sizing the first case (the initial example) to use a single cross-section. In fact, the optimal results with three and four different cross-sections for cases 1, 2, 3, and 4 are roughly about 30–32% lower than this model. Comparing optimal results with 3 and 4 different cross-sections to the analytical result that was produced (699.683 kg), the improvement using optimization is roughly approximately 12–14%, depending on the topology case. It is also interesting that for cases 2, 3, and 4, the global optima are with three different cross-sections, while case 1 has a global optima with four different cross-sections used, and it is only about 4 kg lighter than the result with three cross-sections for that case.

The differences from corresponding optimal solutions, for all four topology cases, show the same trend as the standard truss examples, just with smaller deviations. This is likely due to the roof’s small ranges of sizing and shape variables compared to standard examples. Regardless, all the cases have the same logarithmic difference from the corresponding global optima in this example.

In summary, the findings presented in this study underline the significant impact of cardinality constraints on the optimization of truss design and the potential weight savings when using simultaneous sizing, shape, and topology optimization. By applying the practical constraints, the results demonstrate the practical applicability of this approach, revealing that the global optimal solutions often necessitate a large number of different cross-sections. Through the standard and practical examples and comparative analyses, the results show that the precise cardinality constraints yield insights into optimization trends beyond the global optima, offering a nuanced understanding of optimal design configurations. Moreover, this research highlights the potential benefits of integrating topology optimization, which can lead to substantial material and construction efficiencies in real-world applications. The purpose of these investigations was to set the stage for continued advancements in truss optimization approaches, with implications for enhancing efficiency and sustainability in structural engineering practice.

## Figures and Tables

**Table 1:** HSS profiles are used for the roof truss example and their moments of inertia [17].

Profile Dimensions, mm	Moments of Inertia, cm^4^			
	Profile Wall Thickness, mm			
	3	4	5	6
40 × 40	8.6	11.1	-	-
45 × 45	14.4	17.6	-	-
50 × 50	19.5	23.7	27	-
60 × 60	35.1	43.6	50.5	-
70 × 70	56.1	68.9	-	-
80 × 80	87.8	111	131	149
90 × 90	127	162	193	220
100 × 100	177	226	271	311
110 × 110	235.9	300.3	357.4	439.8
120 × 120	317.2	409.5	498.6	562
130 × 130	397.3	510.2	612.8	748.3
140 × 140	510	661.5	805.8	945.8

**Table 2:** The cross-section areas of optimal models for 10-bar truss sizing, shape, and topology optimization.

Element No.	Cross-Section Areas, ×10^2^ mm^2^								
	Number of Different Cross-Sections Used								
	1	2	3	4	5	6	7	8	9
1	415.476	23.758	181.458	181.458	153.938	132.732	132.732	176.715	201.062
2	-	-	-	-	63.617	78.540	50.265	63.617	78.540
3	415.476	490.874	490.874	490.874	490.874	490.874	490.874	490.874	490.874
4	415.476	490.874	490.874	415.476	314.159	314.159	346.361	346.361	380.133
5	415.476	23.758	28.274	28.274	-	-	-	56.745	12.566
6	-	-	-	-	1.131	1.131	1.131	103.869	103.869
7	-	-	28.274	28.274	63.617	78.540	70.882	63.617	33.183
8	415.476	201.062	-	-	-	-	-	-	-
9	415.476	201.062	181.458	181.458	153.938	201.062	213.825	78.540	63.617
10	-	-	-	-	63.617	78.540	50.265	50.265	1.131
Weight, kg	5896.031	4325.324	3572.348	3386.197	3180.693	3233.19	3268.929	3413.695	3276.435

**Table 3:** Optimal coordinates of points according to the number of different cross-sections used for the 10-bar truss example.

Coordinate	Values, m								
	Number of Different Cross-Sections Used								
	1	2	3	4	5	6	7	8	9
*x*_1_	-	-	-	-	11.585	11.586	11.362	11.406	6.768
*y*_1_	-	-	-	-	1.409	1.387	1.605	4.126	5.569
*x*_3_	9.155	10.304	10.235	9.803	14.15	14.262	13.616	7.619	9.828
*y*_3_	3.628	3.239	3.48	3.749	2.885	2.904	3.231	5.795	4.085

**Table 4:** The cross-section areas of optimal models for 17-bar truss sizing shape and topology optimization.

Element No.	Cross-Section Areas, ×10^2^ mm^2^									
	Number of Different Cross-Sections Used									
	1	2	3	4	5	6	7	8	9	10
1	78.540	56.745	78.540	78.540	78.540	78.540	78.540	78.540	78.540	78.540
2	-	-	-	-	-	28.274	31.172	31.172	44.179	44.179
3	78.540	86.590	78.540	78.540	78.540	86.590	86.590	86.590	95.033	95.033
4	78.540	56.745	28.274	23.758	23.758	50.265	50.265	50.265	44.179	44.179
5	78.540	56.745	56.745	56.745	56.745	63.617	63.617	70.882	86.590	86.590
6	-	-	-	-	-	28.274	31.172	28.274	12.566	12.566
7	78.540	86.590	78.540	78.540	78.540	78.540	78.540	78.540	78.540	78.540
8	78.540	56.745	28.274	31.172	31.172	28.274	38.485	38.485	44.179	44.179
9	78.540	56.745	56.745	56.745	56.745	28.274	31.172	31.172	31.172	31.172
10	-	-	-	-	-	23.758	28.274	28.274	12.566	15.904
11	78.540	56.745	56.745	56.745	56.745	63.617	63.617	63.617	78.540	78.540
12	78.540	56.745	28.274	23.758	23.758	28.274	28.274	28.274	23.758	23.758
13	78.540	56.745	28.274	31.172	31.172	50.265	31.172	31.172	50.265	50.265
14	78.540	56.745	56.745	56.745	56.745	50.265	50.265	50.265	56.745	56.745
15	78.540	56.745	56.745	56.745	56.745	-	-	-	-	-
16	78.540	56.745	56.745	56.745	50.265	-	-	-	-	-
17	78.540	56.745	56.745	56.745	50.265	-	-	-	-	-
Weight, kg	1971.904	1598.321	1390.499	1386.597	1366.206	1303.889	1317.059	1322.443	1376.080	1380.341

**Table 5:** Optimal coordinates of points according to the number of different cross-sections used for the 17-bar truss example.

Coordinate	Values, m									
	Number of Different Cross-Sections Used									
	1	2	3	4	5	6	7	8	9	10
*x*_3_	2.455	2.756	2.541	2.543	2.54	1.985	1.982	2.001	1.976	1.972
*y*_3_	−0.311	−0.146	−0.194	−0.19	−0.187	0.044	0.029	0.061	−0.019	−0.001
*x*_4_	1.668	1.345	1.236	1.247	1.243	3.368	3.334	3.313	3.456	3.456
*y*_4_	2.151	2.419	2.519	2.525	2.525	2.428	2.379	2.375	2.441	2.441
*x*_5_	5.217	5.157	5.202	5.172	5.172	4.791	4.789	4.789	4.772	4.733
*y*_5_	−0.388	−0.01	−0.256	−0.275	−0.371	−0.096	−0.098	−0.099	0.169	0.198
*x*_6_	4.546	4.122	3.765	3.735	3.733	5.861	5.859	5.851	5.618	5.618
*y*_6_	1.55	2.183	2.249	2.232	2.229	1.956	1.956	1.956	2.119	2.082
*x*_7_	7.247	7.638	7.88	7.866	7.857	7.416	7.416	7.415	7.286	7.285
*y*_7_	−0.373	−0.051	−0.328	−0.347	−0.347	−0.006	−0.012	−0.014	0.137	0.12
*x*_8_	6.691	6.762	6.665	6.632	6.632	8.481	8.478	8.474	8.449	8.439
*y*_8_	1.121	1.846	1.846	1.871	1.871	1.647	1.646	1.645	1.514	1.512
*y*_9_	0.52	0.862	0.778	0.747	0.747	0.521	0.52	0.514	0.492	0.458

**Table 6:** The cross-section areas of optimal models for 25-bar truss sizing shape and topology optimization.

Element Group No.	Cross-Section Areas, ×10^2^ mm^2^						
	Number of Different Cross-Sections Used						
	1	2	3	4	5	6	7
1	28.274	-	-	-	-	1.131	2.011
2	28.274	12.566	12.566	1.131	12.566	15.904	9.079
3	28.274	38.485	38.485	38.485	33.183	33.183	33.183
4	-	-	-	-	-	-	1.131
5	28.274	-	-	-	-	-	-
6	28.274	12.566	12.566	4.909	4.909	4.909	9.621
7	28.274	12.566	9.079	28.274	9.621	12.566	12.566
8	28.274	38.485	38.485	38.485	38.485	38.485	38.485
Weight, kg	543.164	392.324	380.098	361.072	328.893	351.122	353.488

**Table 7:** Optimal coordinates of points according to the number of different cross-sections used for the 25-bar truss example.

Node Coordinate	Values, m						
	Number of Different Cross-Sections Used						
	1	2	3	4	5	6	7
−*x*_3_; *x*_4_; *x*_5_; −*x*_6_	0.508	0.932	0.999	0.609	0.995	0.897	0.961
*y*_3_; *y*_4_; −*y*_5_; −*y*_6_	1.245	1.212	1.169	1.4	1.224	1.279	1.168
*z*_3_; *z*_4_; *z*_5_; *z*_6_	2.433	2.472	2.559	2.286	2.59	2.49	2.658
−*x*_7_; *x*_8_; *x*_9_; −*x*_10_	1.016	1.016	1.016	1.016	1.016	1.016	1.016

**Table 8:** Optimal solutions according to the number of different cross-sections used for the first roof truss topology configuration.

Element Groups	Used Standard Profile (Width × Height × Wall Thickness), mm				
	Number of Different Cross-Sections Used				
	1	2	3	4	5
**1–4, 16–19**	90 × 90 × 5	140 × 140 × 3	140 × 140 × 3	140 × 140 × 3	90 × 90 × 5
**5–8, 20–23**	90 × 90 × 5	140 × 140 × 3	140 × 140 × 3	140 × 140 × 3	80 × 80 × 6
**9, 24**	90 × 90 × 5	50 × 50 × 3	45 × 45 × 3	40 × 40 × 3	40 × 40 × 3
**10, 25**	90 × 90 × 5	50 × 50 × 3	45 × 45 × 3	50 × 50 × 3	50 × 50 × 3
**11, 26**	90 × 90 × 5	50 × 50 × 3	40 × 40 × 3	40 × 40 × 3	40 × 40 × 3
**12, 27**	90 × 90 × 5	50 × 50 × 3	45 × 45 × 3	40 × 40 × 3	40 × 40 × 3
**13, 28**	90 × 90 × 5	50 × 50 × 3	45 × 45 × 3	40 × 40 × 3	40 × 40 × 3
**14, 29**	90 × 90 × 5	50 × 50 × 3	40 × 40 × 3	40 × 40 × 3	40 × 40 × 3
**15**	90 × 90 × 5	50 × 50 × 3	40 × 40 × 3	45 × 45 × 3	45 × 45 × 3
**Weight, kg**	879.399	634.235	617.637	613.251	624.138

**Table 9:** Optimal solutions according to the number of different cross-sections used for the second roof truss topology configuration.

Element Groups	Used Standard Profile (Width × Height × Wall Thickness), mm				
	Number of Different Cross-Sections Used				
	1	2	3	4	5
**1–4, 16–19**	90 × 90 × 5	90 × 90 × 5	90 × 90 × 5	80 × 80 × 6	80 × 80 × 6
**5–8, 20–23**	90 × 90 × 5	90 × 90 × 5	90 × 90 × 5	90 × 90 × 5	90 × 90 × 5
**9, 24**	90 × 90 × 5	50 × 50 × 3	40 × 40 × 3	40 × 40 × 3	40 × 40 × 3
**10, 25**	90 × 90 × 5	50 × 50 × 3	45 × 45 × 4	45 × 45 × 3	45 × 45 × 3
**11, 26**	90 × 90 × 5	50 × 50 × 3	40 × 40 × 3	40 × 40 × 3	40 × 40 × 4
**12, 27**	90 × 90 × 5	50 × 50 × 3	40 × 40 × 3	40 × 40 × 3	40 × 40 × 3
**13, 28**	90 × 90 × 5	50 × 50 × 3	40 × 40 × 3	40 × 40 × 3	40 × 40 × 3
**14, 29**	90 × 90 × 5	50 × 50 × 3	40 × 40 × 3	40 × 40 × 3	40 × 40 × 3
**15**	-	-	-	-	-
**Weight, kg**	852.436	629.502	612.018	614.014	616.609

**Table 10:** Optimal solutions according to the number of different cross-sections used for the third roof truss topology configuration.

Element Groups	Used Standard Profile (Width × Height × Wall Thickness), mm				
	Number of Different Cross-Sections Used				
	1	2	3	4	5
**1–4, 16–19**	90 × 90 × 5	90 × 90 × 5	90 × 90 × 5	90 × 90 × 5	90 × 90 × 5
**5–8, 20–23**	90 × 90 × 5	90 × 90 × 5	90 × 90 × 5	90 × 90 × 5	90 × 90 × 5
**9, 24**	90 × 90 × 5	50 × 50 × 3	40 × 40 × 3	40 × 40 × 3	40 × 40 × 3
**10, 25**	90 × 90 × 5	50 × 50 × 3	50 × 50 × 3	50 × 50 × 3	50 × 50 × 3
**11, 26**	-	-	-	-	-
**12, 27**	90 × 90 × 5	50 × 50 × 3	40 × 40 × 3	40 × 40 × 3	40 × 40 × 3
**13, 28**	90 × 90 × 5	50 × 50 × 3	40 × 40 × 3	40 × 40 × 3	45 × 45 × 3
**14, 29**	90 × 90 × 5	50 × 50 × 3	40 × 40 × 3	40 × 40 × 3	40 × 40 × 3
**15**	90 × 90 × 5	50 × 50 × 3	40 × 40 × 3	45 × 45 × 3	40 × 40 × 4
**Weight, kg**	842.164	626.117	606.993	607.891	610.392

**Table 11:** Optimal solutions according to the number of different cross-sections used for the fourth roof truss topology configuration.

Element Groups	Used Standard Profile (Width × Height × Wall Thickness), mm				
	Number of Different Cross-Sections Used				
	1	2	3	4	5
**1–4, 16–19**	140 × 140 × 3	90 × 90 × 5	140 × 140 × 6	90 × 90 × 5	90 × 90 × 5
**5–8, 20–23**	140 × 140 × 3	90 × 90 × 5	140 × 140 × 3	90 × 90 × 5	90 × 90 × 5
**9, 24**	140 × 140 × 3	40 × 40 × 3	40 × 40 × 3	40 × 40 × 3	40 × 40 × 4
**10, 25**	140 × 140 × 3	90 × 90 × 5	60 × 60 × 3	60 × 60 × 3	60 × 60 × 3
**11, 26**	-	-	-	-	-
**12, 27**	140 × 140 × 3	40 × 40 × 3	40 × 40 × 3	40 × 40 × 3	40 × 40 × 3
**13, 28**	140 × 140 × 3	40 × 40 × 3	40 × 40 × 3	45 × 45 × 3	45 × 45 × 3
**14, 29**	140 × 140 × 3	40 × 40 × 3	40 × 40 × 3	40 × 40 × 3	40 × 40 × 3
**15**	-	-	-	-	-
**Weight, kg**	809.798	640.507	601.890	607.306	609.415

**Table 12:** Node coordinates of optimal solutions according to the number of different cross-sections used for all roof truss topology configurations.

Topology Case		Values, m				
	Coordinates	Number of Different Cross-Sections Used				
		1	2	3	4	5
**1**	**−*x*_2_, *x*_11_**	6.81	7.10	7.12	7.10	6.82
	**−*x*_3_, *x*_12_**	5.00	5.01	6.06	5.00	5.00
	**−*x*_4_, *x*_13_**	2.63	2.79	2.66	2.63	2.63
	***y*** **_2–5_** **, *y*_11–13_**	0.80	0.80	0.80	0.80	0.8
**2**	**−*x*_2_, *x*_11_**	−6.81	7.01	6.94	6.81	6.81
	**−*x*_3_, *x*_12_**	5.00	5.00	5.00	5.00	5.00
	**−*x*_4_, *x*_13_**	2.63	2.65	2.54	2.55	2.55
	***y*** **_2–5_** **, *y*_11–13_**	0.8	0.80	0.8	0.8	0.8
**3**	**−*x*_2_, *x*_11_**	6.81	7.01	7.02	7.01	7.00
	**−*x*_3_, *x*_12_**	-	-	-	-	-
	**−*x*_4_, *x*_13_**	2.63	2.69	2.48	2.55	2.59
	***y*** **_2–5_** **, *y*_11–13_**	0.8	0.8	0.8	0.88	0.8
**4**	**−*x*_2_, *x*_11_**	7.02	6.90	7.27	7.15	7.15
	**−*x*_3_, *x*_12_**	-	-	-	-	-
	**−*x*_4_, *x*_13_**	2.63	2.59	2.58	2.54	2.54
	***y*** **_2–5_** **, *y*_11–13_**	0.8	0.87	0.8	0.8	0.8

## Data Availability

The original contributions presented in this study are included in the article. Further inquiries can be directed to the corresponding authors.
